# Diagnostic challenge for ovarian malignant melanoma in premenopausal women: Primary or metastatic?

**DOI:** 10.1186/1477-7819-9-65

**Published:** 2011-06-17

**Authors:** Yassir Sbitti, Zouhour Fadoukhair, Habiba Kadiri, Mohamed Oukabli, Ismail Essaidi, Saoussan Kharmoum, Hind M'rabti, Abderrahmane Albouzidi, Mohammed Ichou, Hassan Errihani

**Affiliations:** 1 Department of Medical Oncology, University Military Hospital, Rabat, Morocco; 2 Department of Medical Oncology, National Institute of Oncology, Rabat, Morocco; 3 Department of Pathology, University Military Hospital, Rabat, Morocco; 4 Department of Pathology, Diagnostic Centre, Rabat, Morocco

**Keywords:** melanoma, ovarian neoplasm, secondary, differential diagnosis

## Abstract

**Background:**

In the ovary, metastatic malignant melanoma may be confused with primary malignant melanoma and presents a diagnosis challenge. Most cases are associated with disseminated diseases and poor prognosis. We present this case report of a metastatic ovarian malignant melanoma simulating primary ovarian cancer.

**Case report:**

A 45-year-old premenopausal woman was incidentally found to have an abdominal mass, 3 years after removal of a cutaneous melanoma lesion. Ultrasound and CT scan revealed left two solid masses, which were found to be an ovarian tumor at laparotomy. Left oophorectomy was performed. Histopathology and immunohistochemistry showed melanoma metastasis to the ovary. Nine months later, the patient developed epilepsy and confusion. Magnetic Resonance Imaging showed unique Wright frontal lobe lesion. She underwent stereotactic radio surgery and dacarbazine monotherapy. For months later, the patient is died from disseminate disease progression.

**Conclusion:**

Ovarian metastasis is an unusual presentation of cutaneous melanoma and the prognosis was dismal. As illustrated by this case report, a differential diagnosis of a metastatic malignant melanoma must be considered.

## Introduction

The ovary is a frequent site of secondary spread from extra-ovarian malignancies. Approximately 6-7% of the patients presenting with suspected ovarian neoplasm will prove to suffer from metastatic disease to the ovary (1). Ovarian involvement by metastatic malignant melanoma is relatively uncommon and it is rare for melanoma to present clinically as an ovarian mass (2). Solitary metastatic malignant melanoma to the ovary may be confused with primary ovarian carcinoma. We present this case report of metastatic ovarian malignant melanoma simulating primary ovarian cancer.

## Case report

**Figure 1:**
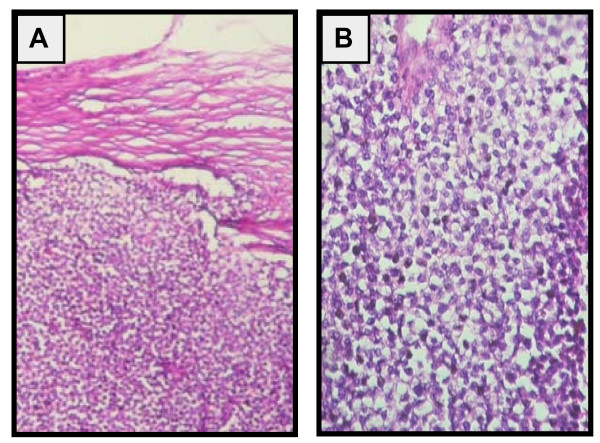
**Diffuse pattern of round cells**. (A) contains abundant cytoplasm, round to ovoid nucleus and prominent nucleoli (B). A: original magnification × 40 H&E. B: original magnification × 200 H&E.

**Figure 2:**
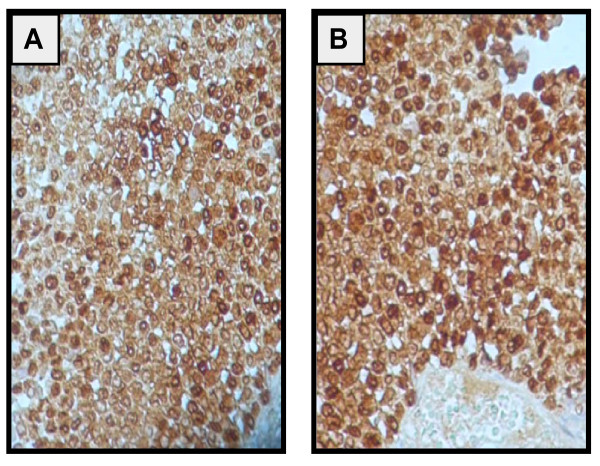
**A: PS100 is strongly expressed in both the nucleus and the cytoplasm**. Original magnification × 400 (PS100) B: Immunostain for Melan A shows a diffuse, intensively cytoplasmic positive reaction. Original magnification × 200 (Melan A)

In February 2010, a 45 years old premenopausal female patient presented to us because acute pelvic pain in relation with left ovarian mass. She gave no significant gynecologic history. Abdominal and gynaecologic exam disclosed left adnexal mass. Abdominal ultrasound completed with computed tomography (CT) scan revealed two solid irregular masses measuring respectively 84 × 46 mm, 45 × 34 mm, located centrally in the pelvis and appearing to originate from the left side of the uterus. Thorax (CT) scan and biologic laboratory investigations were normal. The tumor marker, CA125, and levels of CEA (carcinoembryonic antigen), AFP (alpha feto-protein) and beta-HCG (human chorionic gonadotrophin) were normal. Previous history revealed a management for a left plantar melanoma with palpable inguinal lymph node without skip metastasis in December 2006. Preoperative thoraco-abdominal and pelvic CT scan and cerebral magnetic resonance imaging were normal. Local excision lesion and elective inguinal lymph node dissection were performed. Histopathology study showed a superficial spreading melanoma with a nodular phase growth pattern, breslow index 1,7 mm and Clark's (VI) with 2,5 cm free surgical margins. 3 out of 8 lymph nodes dissected involved by metastatic melanoma. The patient received adjuvant interferon alpha high dose for eighteen months at medical oncology department. Follow-up of the patient for the ensuing 3 years was without any signs of recurrence. At laparotomy, a left ovarian mass measuring 15 cm with an intact and smooth capsule without adhesions to its surroundings was discovered, whereas the right ovary and uterus grossly appeared normal. Infracolic-omentum and peritoneal surface were free of peritoneal nodule. Left salpingo-oophorectomy, right ovarian biopsy and uterine biopsy via hysteroscopy were performed. Preleved tissue was sends for frozen section examination. Histopathology analysis of left ovarian mass objective a surgical specimen measured 11 by 9.5 by 5 cm and weighed 320 g. Cut surface showed a multilocular cyst containing white and cerebriform intracystic vegetations, with necrotic hemorrhagic foci. The neoplasm formed diffuse sheets. The fragments presented a diffuse infiltrate, masking the normal tissue. This infiltrate consisted of a monomorphous proliferation of large cells, with an abundant pale or eosinophilic cytoplasm. The nuclei were large round or oval, and contained prominent nucleoli. Mitotic figures were rare. In many places, the tumour had been destroyed by large areas of ischemic necrosis. The proliferation was supported by a thin delicate fibro vascular stroma. (Figure 1). Right ovarian and uterine biopsies were intact. No melanin pigment was identified in the tumor Cells. In the immunochemistry, Inhibine, CD99, EMA, chromogranin, synaptophysin were negative; the tumour cells strongly expressed PS100 and Melan A (Figure 2A) HMB45 (Figure 2B) was observed in some cells suggesting the diagnosis of metastatic ovarian amelanic melanoma. Postoperative Thoracoabdominopelvien CT scan was normal. Dacarbazine was proposed for patient but she refused to receive it. After nine month of pelvic surgery and disease stabilization, the patient developed confusion and epilepsy. Magnetic Resonance Imaging showed a unique brain metastasis lesion in the Wright frontal lobe. She underwent stereotactic radio surgery followed by Dacarbazine chemotherapy. The patient died 4 months later.

## Discussion

Melanoma affects usually the skin, adrenal glands and ocular choroids with frequent sites of metastasis to the skin, brain and lung and rarely to the ovary [1]. However, once the diagnosis of malignant melanoma of the ovary has been established; the distinction of a primary ovarian malignant melanoma from a metastatic tumor must be made. Some authors [2,3] have restricted the diagnosis of primary ovarian malignant melanoma to those cases with no history of malignant melanoma at any other site, where the tumor is confined to the ovary, and where it arises in association with ovarian cystic teratomas. Metastatic ovarian malignant melanomas are more common than primary ovarian malignant melanomas; to date, about 77 cases of malignant melanoma metastatic to the ovary [4-7] compared to only about 31 cases of primary ovarian malignant melanoma including a compilation of 20 cases and individual case reports [8-11] have been reported in the world literature. Although approximately 20% of patients dying of malignant melanoma have ovarian involvement at postmortem examination, the diagnosis of ovarian malignant melanoma is seldom made before autopsy [12]. This is because most of these patients have multiorgan metastases so the involvement of the ovary is not clinically significant. However premortem diagnosis is uncommon and sufficiently symptomatic to be diagnosed in living patients. The time interval between the diagnosis of the primary melanoma and ovarian metastasis has ranged from months up to 18 years [13]. Our patient had an intermediate, stage III melanoma, thus a 36% risk of death at 10 years [14]. She developed ovarian mass three years after melanoma diagnosis. Ovarian metastases from melanoma are mostly unilateral [13], as in our patient. Women of reproductive age are more prone to metastatic ovarian involvement, which may be attributed to the higher blood flow to the premenopausal ovary [12]. The extremities are the most frequent primary localization of melanoma, secondarily involving the ovaries [13], as in our patient. Metastatic melanoma to the ovary can mimic a primary ovarian malignancy and may pose a diagnostic challenge. The majority of ovarian metastasis from melanoma published so far has been almost invariably diagnosed following surgical treatment [4,13,15,16]. Levels of tumor markers are non discriminatory and Ultrasound and CT scan were unable to characterize the lesion as in our patient [15]. However Magnetic resonance imaging of the lesion can raise suspicion of its nature because of a hyper-intense signal in T1-weighted images. This hyper-intense signal is related to the amount of melanin in the lesion and is finally present in about a third of cases [15,16]. In our patient, the possibility of the cutaneous melanoma metastasizing to the ovary was not considered being very rare and MRI was not performed. The pathological diagnosis is also difficult, since the morphology of the lesion is often nonspecific. An achromic or poorly pigmented lesion often leads to several biopsies before the tumor is clearly defined. Diagnostic difficulties also arise histologically as the tumors do not have a consistent appearance and they can be mistaken for germ cell and sex cord stromal tumors or granulosa tumor [13]. Hence, definite diagnosis relies on immunohistochemistry. S-100 is expressed in both the nucleus and the cytoplasm and has been found to be the most sensitive marker, present in 95% of cases [3,5-7]. HMB-45 and Melan A are expressed in the cytoplasm. In our patient's, history of cutaneous melanoma, the absence of cystic teratomas lesion and tumor positive stain for PS100 and Melan A markers (Figure 2) and negative stain for of alpha-inhibin and CD99 confirmed the diagnosis of metastatic ovarian melanoma. The management of ovarian tumor is surgical, with removal of the tumor and evaluation for local, regional and distant spread. Initial staging should evaluate thoroughly disease extent and obtain pathologic diagnosis to guide further treatment. Metastatic melanoma is associated with poor prognosis with 11% 5-year survival [14]. Surgical treatment for abdominal metastases of melanoma in one report significantly prolonged survival; however complete resection was only possible in one-third of the patients [17]. Unilateral salpingo-oophorectomy has been proposed as an appropriate treatment for metastatic melanoma involving the ovary, if there is no evidence of controlateral ovarian involvement or extra ovarian spread [4,12]. In such cases of apparently resectable metastatic disease, preoperative screening for metastatic disease in other sites is crucial, either with conventional imaging or with PET scanning [18]. In disseminated diseases, chemotherapy (single-agent or combined) generally only yields a response rate of up to 25%. Though early studies of bio-chemotherapy and immunotherapy were promising, most randomized trials have failed to demonstrate a significant increase in response rate or overall survival.. More targeted approaches that capitalize on recent advances in our understanding of melanoma pathogenesis have emerged. Pro-apoptotic agents 'Oblimersen', an antisense inhibitor of Bcl-2, and 'Sorafenib', an orally active small molecule inhibitor of wild type and mutant BRAF or PLX4032 a potent inhibitor of BRAF with the V600E mutation are at the forefront of novel therapies developed for advanced metastatic disease [19,20]. In our case the no evidence of right lesion and abdominal spread, left unilateral oophorectomy was performed without adjuvant chemotherapy. The patient had a good postoperative recovery with good health at nine months. She developed unique cerebral metastases lesion treated with radio surgery followed by dacarbazine. 4 months later, the patients died from disseminate disease progression.

## Conclusion

In conclusion, this case illustrate that ovarian melanoma is a rare disease, associated with a poor prognosis. Also imaging modalities may be non conclusive and immunohistochemistry must be relied upon to make the final definitive diagnosis. Metastatic presentation is more common than primary ovarian melanoma and should be suspected in any patient who presents a history of malignant melanoma with an ovarian mass without benign cystic teratomas, even if the remission period is long and the ovary is the initial site of relapse. Surgical interventions may be undertaken only in limited metastatic disease, where complete resection is expected.

## Consent

Written informed consent was obtained from the patient's husband for publication of this case report and any accompanying images.

## Competing interests

The authors declare that they have no competing interests.

## Authors' contributions

YS - ZF performed literature review, the composition of this case report and manuscript writing. NI-HK conception and design collection and assembly of data. AA - MO performed histopathologic analysis and obtain photomicrographs. MI- HE analyse and interpretation of data, manuscript writing. All authors read and approved the manuscript.
